# Acceptability of a Digital Adherence Tool Among Patients With Tuberculosis and Tuberculosis Care Providers in Kilimanjaro Region, Tanzania: Mixed Methods Study

**DOI:** 10.2196/51662

**Published:** 2024-06-26

**Authors:** Alan Elias Mtenga, Rehema Anenmose Maro, Angel Dillip, Perry Msoka, Naomi Emmanuel, Kennedy Ngowi, Marion Sumari-de Boer

**Affiliations:** 1 mHealth Department Kilimanjaro Clinical Research Institute Moshi United Republic of Tanzania; 2 Department of Life Science and Bioengineering Nelson Mandela African Institution of Science and Technology Arusha United Republic of Tanzania; 3 Department of Health system Apotheker Health Access Initiatives, Tanzania Dar es salaam United Republic of Tanzania

**Keywords:** acceptability, digital adherence tool, medication reminder monitors, patients with tuberculosis, TB, adherence, TB care provider

## Abstract

**Background:**

The World Health Organization has recommended digital adherence tools (DATs) as a promising intervention to improve antituberculosis drug adherence. However, the acceptability of DATs in resource-limited settings is not adequately studied.

**Objective:**

We investigated the acceptability of a DAT among patients with tuberculosis (TB) and TB care providers in Kilimanjaro, Tanzania.

**Methods:**

We conducted a convergent parallel mixed methods study among patients with TB and TB care providers participating in our 2-arm cluster randomized trial (REMIND-TB). The trial aimed to investigate whether the evriMED pillbox with reminder cues and adherence feedback effectively improves adherence to anti-TB treatment among patients with TB in Kilimanjaro, Tanzania. We conducted exit and in-depth interviews among patients as well as in-depth interviews among TB care providers in the intervention arm. We conducted a descriptive analysis of the quantitative data from exit interviews. Translated transcripts and memos were organized using NVivo software. We employed inductive and deductive thematic framework analysis, guided by Sekhon’s theoretical framework of acceptability.

**Results:**

Out of the 245 patients who completed treatment, 100 (40.8%) were interviewed during exit interviews, and 18 patients and 15 TB care providers were interviewed in-depth. Our findings showed that the DAT was highly accepted: 83% (83/100) expressed satisfaction, 98% (98/100) reported positive experiences with DAT use, 78% (78/100) understood how the intervention works, and 92% (92/100) successfully used the pillbox. Good perceived effectiveness was reported by 84% (84/100) of the participants who noticed improved adherence, and many preferred continuing receiving reminders through SMS text messages, indicating high levels of self-efficacy. Ethical concerns were minimal, as 85 (85%) participants did not worry about remote monitoring. However, some participants felt burdened using DATs; 9 (9%) faced difficulties keeping the device at home, 12 (12%) were not pleased with receiving daily reminder SMS text messages, and 30 (30%) reported challenges related to mobile network connectivity issues. TB care providers accepted the intervention due to its perceived impact on treatment outcomes and behavior change in adherence counseling, and they demonstrated high level of intervention coherence.

**Conclusions:**

DATs are highly acceptable in Tanzania. However, some barriers such as TB-related stigma and mobile network connectivity issues may limit acceptance.

**International Registered Report Identifier (IRRID):**

RR2-10.1186/s13063-019-3483-4

## Introduction

Tuberculosis (TB) is a significant public health problem and the second leading infectious killer after COVID-19 [1]. The World Health Organization has set a target in its “end TB strategy” to reduce TB deaths by 75% in 2025 and 90% in 2030 [2]. Tanzania is among the 30 countries with high TB burden and is estimated to have had a TB incidence of 208 per 100,000 persons and 1.3% of multidrug-resistant TB cases in 2021 [1]. In 2020, Tanzania reported that about 26,800 people died from TB [3]. TB is a curable disease if adequate treatment is implemented [4]. However, treatment adherence is a major challenge that hinders TB treatment efforts [5]. Insufficient adherence to TB medication is contributed by multiple factors such as the social context, health system, economic factors, patient-related factors (forgetfulness, low knowledge), health service accessibility, and drug-related factors such as drug side effects [6,7].

The World Health Organization has recommended digital adherence tools (DATs) that include SMS text messages, medication event monitoring devices, and video-observed treatment as promising interventions for improving TB adherence [8]. DATs can remind patients to take their medications, offer dose information, alert health care practitioners to risky behavior patterns, and allow health care practitioners to intervene when treatment is interrupted [9]. Furthermore, digital devices provide baseline information for health care providers during adherence counseling, patient motivation, replacement of interrupted medication, and scheduling clinic visits [8].

DATs have proven feasible in high- and low-resource settings [9-11]. Further, studies have shown that these devices have relatively high acceptability in Tanzania among people living with HIV, in Uganda and China among patients with drug-susceptible TB, and in South Africa among patients with multidrug-resistant TB [12-15]. However, literature indicates that the wide implementation of DATs in China has shown challenges, such that 11.3% refused to use DATs at enrollment and 8.2% refused to use DATs during treatment [16]. Another study in Vietnam showed that participants could not use the pillbox as required because they could not carry it to their workplace [17]. More evidence on the acceptability of DATs is needed to inform its large-scale implementation in resource-limited settings.

Investigating implementation research outcomes such as acceptability, feasibility, sustainability, and adoption is essential for identifying implementation bottlenecks that may hamper intervention effectiveness in a real-world setting [18]. In addition, when a health care intervention is not considered acceptable, it may affect health care providers’ perception and treatment delivery [19]. In this study, we aimed to investigate the acceptability of DATs among patients and TB care providers for improving adherence to anti-TB drugs among patients with TB in Kilimanjaro, Tanzania.

## Methods

### Study Design

We conducted a convergent parallel mixed methods study, which was embedded in our cluster randomized trial (REMIND-TB), among patients with TB and TB care providers.

### Ethics Approval

This study was approved by the Kilimanjaro Christian Medical College research ethics and review committee (approval 1157, dated December 10, 2018) and the National Health Research ethics subcommittee (ref NIMR/HQ/R.8a/VolIX/2992, dated January 14, 2019). We registered the trial at the Pan African Clinical Trials Registry under PACTR201811755733759 on November 8, 2018.

### REMIND-TB Trial

From 2019 to 2021, we conducted a 2-arm cluster randomized trial to investigate whether the evriMED pillbox with reminder cues and adherence feedback effectively improved adherence to anti-TB treatment among patients with TB in Kilimanjaro region in Tanzania. Study sites were randomized into 12 clusters: 6 intervention arms and 6 control arms. The inclusion criteria for the trial were patients’ diagnosis with presumptive sensitive TB, aged 18-65 years, attending care at any of the TB centers in the Kilimanjaro region of Tanzania, willing to use the evriMED pillbox, able to read and understand SMS text messages, and able to understand and willing to sign informed consent. Exclusion criteria were hospitalized patients and those who previously participated in similar studies. We provided each participant in the intervention arm with an evriMED1000 pillbox for their medication storage and intake. In the control arm, participants followed the standard of care procedures. In both arms, we followed participants for 6 months of treatment. In the evriMED arm, participants received a reminder SMS text message every day 30 minutes before their set time of taking medication. Detailed information about the REMIND-TB trial and the DAT can be found elsewhere [20].

### evriMED Pillbox

The evriMED1000 is a type of tablet dispenser with a SIM card produced by Wisepill, based in South Africa. The pillbox records the opening of the box and stores the so-called medication events on a chip, along with the date and time whenever it is opened. This information is transmitted to a centralized server when one opens the device. Additionally, the evriMED1000 delivers a daily heartbeat event that includes information about the device’s identification, battery life, and signal quality. If the pillbox is not opened on a particular day, any pending events will be transmitted during the next heartbeat.

The evriMED1000 sends a reminder SMS text message to an individual’s mobile phone 30 minutes before intake time. If the individual does not take the medication within 1 hour of the intake period, a second reminder SMS text message is generated and sent. The patient does not require internet connectivity to receive the reminder SMS text message. The Wisepill pillbox uses a global roaming SIM chip that will connect to the best available mobile network in the area. These devices are designed to work in low-network resource settings (see Multimedia Appendix 1).

As the trial was an implementation study, TB care providers took full responsibility for participant care within their regular duties. They had to explain and demonstrate the use of the evriMED device to patients, provide medication through the device, and discuss adherence reports generated by the device during follow-up clinics. Participants were trained on device usage upon enrollment and were required to stay with it for the entire 6-month treatment period. Any challenges related to device functionality or misunderstandings were addressed through ongoing discussions between patients and care providers during follow-up visits.

### Mixed Methods Study on Acceptability of DATs

#### Study Procedures

Enrolled participants from the intervention arm of the REMIND-TB trial who completed 6 months of treatment were called for a phone interview. One inclusion criterion for this study was that participants had to be randomized into the intervention arm, while we excluded all the participants randomized into the control arm. The other inclusion and exclusion criteria were the same as in the trial. In addition, we purposively selected 18 patients with TB for an in-depth interview. Considering that this was an implementation study in which all the activities were performed by TB care providers, we purposively selected 15 TB care providers to understand the acceptability pattern of evriMED. We aimed at a heterogenic sample for both patients and health care workers by considering patients with good and poor adherence as well as diverse professional experiences among TB care providers. We obtained written informed consent from all individuals who participated in the acceptability study. After completing the follow-up of all participants in the REMIND-TB trial, we purposively selected TB treatment centers with their respective care providers in each cluster, who were called for an in-depth interview. Interviews were performed by trained research assistants using a topic guide in the Swahili language. Audio recordings of the interviews were transcribed and translated by experienced research assistants.

#### Theoretical Framework of Acceptability

The theoretical framework of acceptability (TFA) is a theoretical framework that helps to evaluate the intervention acceptability based on the lived or perceived experiences of individuals who deliver or receive an intervention [19]. The TFA has 7 constructs that can evaluate acceptability before, during, and after implementation performance (Table 1). In this study, we used TFA to investigate the acceptability of DATs among patients with TB and TB care providers. We believe that this theoretical framework is the best to use for this study due to its robustness in integrating the comprehensive concept of acceptability derived from diverse theories in health psychology and behavior change. To our knowledge, this inherent strength makes TFA the best theory of acceptability when compared to other theories. In addition to that, several studies have employed this theory to evaluate the acceptability of health care interventions [15,21-23].

**Table 1:** Constructs and description of the theoretical framework of acceptability.

Constructs	Description
Affective attitude	How an individual feels about the intervention
Burden	The perceived amount of effort that was required to participate in the intervention
Ethicality	The extent to which the intervention has a good fit with an individual’s value system
Intervention coherence	The extent to which the participant understands the intervention and how it works
Opportunity costs	The extent to which benefits, profits, or values were given up to engage in the intervention
Perceived effectiveness	The extent to which the intervention is perceived to have achieved its intended purpose
Self-efficacy	The participant’s confidence that they can perform the behavior(s) required to participate in the intervention

### Data Collection Tools

#### Exit Survey

We conducted an exit survey after a participant completed the treatment. The survey was conducted by phone by trained research assistants. We performed the survey by using a semistructured questionnaire that we developed using the 7 constructs of the acceptability framework (affective attitude, perceived burden, ethicality, perceived effectiveness, intervention coherence, self-efficacy, and opportunity cost).

#### In-Depth Interviews

We conducted in-depth interviews with patients and TB care providers from the intervention clusters. Patients and TB care providers were interviewed at their respective health facilities at the agreed time. All interviews were conducted by 2 experienced researchers led by the first author (AEM). We used different topic guides for in-depth interviews with patients and care providers, respectively. We used the Sekhon framework for acceptability to define the guide [19]. Questions mainly focused on the 7 constructs of TFA. The questionnaires were adapted if new topics came up during interviews.

### Data Analyses

To answer the objective regarding the acceptability of evriMED among patients with TB and TB care providers, we conducted a descriptive analysis of the exit survey responses using STATA (version 15; Stata Corp LLC). The results of the exit survey provided an overview of the frequency and percentages of each element of acceptability. In addition, we analyzed the qualitative responses of participants and TB care providers inductively and deductively by using thematic framework analyses. Three researchers independently read the transcripts (AEM, MSdB, and RAM). We developed memos and subthemes inductively based on the narratives and deductively by adopting preidentified themes from the theoretical framework constructs. We uploaded transcripts and memos in NVivo software (Lumivero) for coding and data organization. Narratives from the transcripts were then coded based on the predefined subthemes.

## Results

### Description of the Patients

We enrolled 280 patients in the intervention arm; of these, 21 (7.5%) died before study completion, and 14 (5%) were excluded due to either being transferred to other regions or lost to follow-up. Of the 245 (87.5%) patients who completed treatment, 145 (59.2%) were not interviewed because their phone numbers were unreachable. We interviewed 100 (40.8%) patients. The details of the demographic characteristics of the patients with TB are shown below in Table 2.

In addition, we in-depth interviewed 18 patients and 15 TB care providers or directly observed therapy, and we reached data saturation in these interviews. Among the 18 patients, 12 (67%) were males and 6 (33%) were females. Detailed demographic characteristic are shown in Table 3. Of the 15 TB care providers who were interviewed, 3 (20%) were males and 12 (80%) were females, of whom 4 (27%) were clinicians, 1 (6%) was a pharmacist, 4 (27%) were medical attendants, and 6 (40%) were registered nurses.

**Table 2:** Demographic characteristics and treatment outcomes of the patients with tuberculosis (N=280).

Characteristics			Values, n (%)
**Sex**			
	Male	207 (73.9)	
	Female	73 (26.1)	
**Inclusion clusters**			
	Moshi rural district hospital	21 (7.5)	
	Moshi rural health center	115 (41)	
	Moshi urban district hospital	3 (1.1)	
	Moshi urban health center	42 (15)	
	Same and Mwanga health facilities	49 (17.5)	
	Kibongoto National Infectious Disease hospital	50 (17.9)	
**Age (years)**			
	<20	2 (0.7)	
	20-29	50 (17.9)	
	30-29	57 (20.4)	
	40-49	89 (31.8)	
	50-59	52 (18.6)	
	≥60	30 (10.7)	
**Education level**			
	None	9 (3.2)	
	Primary	213 (76.1)	
	Secondary	56 (20)	
	Tertiary	2 (0.7)	
**Marital status**			
	Married	155 (55.4)	
	Single	79 (28.2)	
	Separated or divorced	35 (12.5)	
	Widowed	11 (3.9)	
**Treatment outcome**			
	Cured/completed treatment	245 (87.5)	
	Transferred and lost	14 (5)	
	Dead	21 (7.5)	

**Table 3:** Demographic and adherence characteristics of the in-depth interviews with individuals diagnosed with tuberculosis.

	Sex	Age (years)	Education	Marital status	Participant’s adherence as shown by DAT^a^ (%)^b^
1	Female	30	Secondary	Married	10
2	Male	40	Primary	Married	24
3	Male	58	Primary	Married	21
4	Male	55	Primary	Married	38
5	Female	53	Primary	Single	99
6	Male	48	Primary	Married	99
7	Male	56	Primary	Married	21
8	Female	38	Primary	Single	0
9	Female	49	Primary	Married	100
10	Male	63	Secondary	Married	100
11	Female	45	Primary	Separated	68
12	Male	52	Primary	Married	90
14	Male	59	Primary	Married	99
14	Male	41	Primary	Married	99
15	Female	45	Primary	Divorced	41
16	Male	52	Primary	Married	93
17	Male	40	Primary	Married	96
18	Male	60	Secondary	Married	98

^a^DAT: digital adherence tool.

^b^Absolute values of the percentages are not provided because the adherence score in the evriMED monitor is automatically generated by the pillbox based on the patient’s daily medication intake behavior.

### Patients’ and TB Care Providers’ Acceptability of DAT

The table summarizing the quantitative survey findings can be found in Multimedia Appendix 2.

#### Affective Attitude

Many participants described positive views concerning the use of the intervention. In the exit interview, 98% (98/100) of the participants indicated their general experience with the pillbox was either good or very good, 83% (83/100) reported that the intervention was satisfactory, and 85% (85/100) had either a good or very good attitude toward the content of the reminder SMS text messages. Of the 20 people who saw their adherence graphs, 18 (90%) had a good or very good attitude toward graphs.

In the in-depth interviews, participants expressed positive opinions about the appearance and attractiveness of the pillboxes. They were particularly impressed with the white color of the pillbox. TB care providers and patients acknowledged the appropriateness of the pillbox’s size, stating that it allowed for hygienic medication storage. However, some TB care providers suggested increasing the pillbox size to accommodate patients’ cards. Furthermore, some participants appreciated the pillbox’s size as it matched the size of TB drug blisters. Additionally, participants with comorbidities (TB-HIV) found the large size of the pillbox advantageous for storing drugs for other diseases. This is illustrated in the following quotes.

…From my point of view, the device is good. Even from looking at it. Even the color itself is not bad.

Patient, 42-year-old male

…it reminds him. Even though he doesn’t have his phone, it helps him think he should take medication. The second thing I see is that drugs stay safe. Thirdly, it helped patients to be alert. They were swallowing the medicine on time, and if they forgot, it reminded them.

TB directly observed therapy, registered nurse

Participants and TB care providers highlighted the benefits they experienced from using the intervention, particularly regarding medication reminders and storage. TB care providers expressed satisfaction with attending to patients utilizing the intervention, as it enabled them to monitor progress through adherence reports. However, a few participants expressed negative sentiments. One participant suggested that having pillboxes in different colors would be more attractive, as the white color could quickly get dirty. Another participant felt the pillbox size was too large to carry and recommended reducing its size by half, as also mentioned by some other participants.

…It alerted us that why this guy/patient has this problem. Let us call him and sit to talk with him about what the problem is.

TB directly observed therapy, medical attendant

…The first advantage is to be reminded. You understand me. It reminds you. I have been reminded many times because I also like to sleep; if I do not go out, I always like to sleep at home. Also, drug storage.

Patient, 30-year-old female

…It’s good, but when it’s new. If it is new, it is very attractive. Now, it shows it has been used. It is clean, but not attractive anymore.

Patient, 56-year-old male

…I don’t know…the size should be reduced to half! I see it is big.

Patient, 30-year-old female

#### Perceived Burden

We examined the perceived effort involved in using the intervention. During the exit interview, some participants (n=100) faced challenges when using the intervention. Specifically, 10 (10%) respondents mentioned experiencing TB-related stigma, 12 (12%) expressed discomfort with receiving daily SMS text message reminders, 7 (7%) found it challenging to use the device, 9 (9%) encountered difficulties keeping it at home, and 3 (3%) reported issues with charging the device. Additionally, 30 (30%) reported experiencing challenges with mobile network connections.

In-depth interviews revealed a few aspects that participants and TB care providers were experiencing in using the intervention. Few participants expressed challenges in travelling with the pillbox. TB care providers mentioned that the intervention increased their workload, as it required extensive discussions with patients about various aspects of adherence. Moreover, mobile network–related issues caused delays in the system’s signal transmission when the pillbox was opened, leading to poor adherence reports for some patients and incorrect SMS text message notifications. Some participants suggested that the system should not send reminder SMS text messages to treatment supporters, as the device occasionally failed to detect events due to network problems. This can be seen from the following quotes.

…I did not feel comfortable going with it because others would suspect me [of being sick].

Patient, 56-year-old male

…But the time was insufficient according to the working environment. So, once you get a patient in this environment, it is a bit of a challenge to sit with them. You must be brief because the time is insufficient, and you might need to work in the OPD wards simultaneously. So, if you sit with that patient for a long time, you will cause a jam in another unit.

TB directly observed therapy, medical attendant

…If the network is fine, the adherence is good. But, if he goes to a place without a network, the device is not communicating even if he has taken the medicine.

TB directly observed therapy, medical attendant

#### Ethicality

Many participants and care providers described the intervention as fitting well with their value system. Exit interviews with participants revealed that 85% (85/100) did not worry about being monitored remotely, and 77% (77/100) said they did not experience any form of stigma. Similar findings were observed in the in-depth interviews. Many participants considered the pillbox morally acceptable and appreciated how it helped maintain their confidentiality. TB care providers also found the content of the reminder SMS text messages to be beneficial for their patients, as illustrated in the quotes below.

…I saw the benefits of hiding the secret of my illness. The device is acceptable for my side. I do not know for others.

Patient, 56-year-old male

…It is morally right to use the device.

Patient, 42-year-old male

…I think the SMS contents were fine.

TB directly observed therapy, medical attendant

Furthermore, participants emphasized that the pillbox and SMS text messages aligned with their social values within their families. They highlighted that the intervention facilitated ongoing support from their families throughout the medication period, as described by the following participants.

…even my wife told me: “The time to take the medicine is near. Go and take the medicine.” Even if the hours have not arrived, she remembers.

Patient, 42-year-old male

…They supported me well. For example, giving me milk food. Even, sometimes, when they do cleaning, they wipe the device.

Patient, 48-year-old female

#### Intervention Coherence

Most participants and TB care providers claimed to understand the intervention and how it works. The findings from the exit interview revealed that 78% (78/100) of the respondents indicated they understood the intervention, 92% (92/100) mentioned they could use the intervention without any challenge, and 84% (84/100) could charge the device without problems. However, only 20% (20/100) of the participants were shown their adherence graphs during their counseling sessions with care providers.

Similar findings emerged during the in-depth interviews, where many participants and TB care providers effectively communicated the purpose of the intervention and its operational processes. During the interview, we asked health care providers to show how they had informed participants about intervention objectives and how it worked. The in-depth interviews revealed that health care workers understood the intervention’s objectives and were adept at conveying this information to their patients. Furthermore, participants and care providers were able to explain how different components of the pillbox, such as the alarm, lights, charging system, and reminder system, communicate with the server. However, the interviews revealed that most participants did not remember the name of the pillbox. Instead, they used to call it by their local name, kiboksi, which means “the box.” This is demonstrated in the following quotes.

…It reminds you to swallow the medicine, so when you open it, it indicates someone has opened the device and swallowed it. When you do not open it, it means you have not swallowed it. So, you will be sent a reminder message.

TB directly observed therapy, registered nurse

…This device, first, is the one we use to store medicine. Second, when you open this device, it turns on the lights and gives an alarm. Once you have taken out the medicine inside and used the one you need, the other ones you must put back in. When you put them back inside the device, you close this device. If you close it properly, the lights turn off. One thing I have noticed is that it gets to the point where you open it, and then the lights turn on and off. The moment it turns on and off, it does not show the indicator again.

Patient, 63-year-old male

…Honestly, the graph has never been shown to me.

Patient, 30-year-old female

#### Perceived Effectiveness

Participants and TB care providers expressed that the intervention successfully achieved its intended goal. In the exit interviews, 84% (84/100) of the respondents acknowledged that the intervention improved their treatment adherence. Similar findings were observed in the in-depth interviews with both participants and TB care providers. Participants mentioned that the intervention facilitated adherence by providing timely reminders, enabling them to stick to their scheduled intake times. They found the SMS text message reminders especially helpful when occupied with other activities and when prone to forgetfulness. Furthermore, TB care providers reported that the intervention significantly improved the treatment outcomes for patients compared to those who did not use the pillbox, as described in the following quotations.

…Receiving the message that says “the time of intake is near.” That has helped me a lot because you are probably far from home. So, you will estimate I have 20 minutes or half an hour to be home.

Patient, 63-year-old male

…I thank God, to be honest, no patients could stop medication or even die.

TB directly observed therapy, medical attendant

…Honestly, I have been successful because many patients have recovered; they didn’t get resistance.

TB directly observed therapy, clinician

Health care providers expressed that the intervention improved their rapport with patients by providing feedback on adherence counseling, fostering a sense of compassion and love. They found the adherence report valuable in effectively monitoring the patients’ progress. Moreover, the intervention resulted in positive behavioral changes among TB care providers. Many providers mentioned that the feedback in adherence counseling sessions helped them refine their approach when attending to patients with TB, and they gained a better understanding of the significance of adherence in time of medication, which had previously been given less attention, as described in the following quotations.

…It helps to keep the closeness…among the patients…You even get time to talk to him and discover what’s happening with him. Many positive patients have come out completely healed.

TB directly observed therapy, registered nurse

…For us care providers, it was helpful because we are not doing one work, but also doing other work. So, once we get the patient’s information for reference from the devices, it helps us to know if the patients are in good care compared to those who are not using the devices.

TB directly observed therapy, medical attendant

…in the past, we were giving drugs, but we did not emphasize that if a patient should swallow medicine at 8 AM, it should be taken at 8 AM every day. We used to tell them to take drugs in the morning regardless of the time. For this study, we dispensed and told them to choose whether it was 8 or 9 o’clock. He will choose and should take the drug at the same time every day. And we have seen that it has brought great success.

TB directly observed therapy, clinician

#### Opportunity Costs

Few participants mentioned that they had to give up something valuable to participate in this study. Exit interview results show that 6% (6/100) of the respondents incurred extra costs while using the intervention. From the in-depth interviews, 1 participant expressed that he incurred higher costs because he received a reminder when he was away from home and did not want to ruin his intake report. Therefore, he decided to take a quick transport to get home on time. Another participant mentioned working fewer hours than usual to get home early to take medication on time.

…There was a period when I was receiving messages, but if I went somewhere and became late, I had to take a quick motorcycle.

Patient, 63-year-old male

…That happens once in a while because you may find that you are working somewhere, and then you still have time, but you have to leave early.

Patient, 42-year-old male

#### Self-Efficacy

Many participants and TB care providers said they were confident to engage in the intervention. In the exit interviews with participants, 84% (84/100) of the respondents said they were comfortable to continue receiving reminder SMS text messages every day. The same was reported in the in-depth interviews, in which some of the participants expressed that they preferred the device to be given to many patients and not to a few just for research. Others mentioned that they were confident in explaining the pillbox to their families and relatives. TB care providers expressed that the intervention would be suitable to be adopted in their care and, if possible, include patients experiencing other diseases such as HIV, as illustrated in the quotations below.

…For my part, I advise this research project to continue. Not just for research purposes only and end there. It should continue because it is a good thing, and the scope should be expanded to get more people to use this device.

TB directly observed therapy, registered nurse

…This device is so good to the extent that I liked it and wished I could remain with it.

Patient, 53-year-old male

## Discussion

### Principal Findings

This study aims to evaluate the acceptability of a DAT (evriMED1000 pillbox) among patients and TB care providers to improve adherence to anti-TB drugs in Kilimanjaro, Tanzania. The overall findings of this study indicate high acceptance of DAT among patients with TB and TB care providers. We found that the high acceptance of DATs was based on the positive attitude toward using the DAT (affective attitude), wherein 83% (83/100) of the participants were satisfied with the intervention, 98% (98/100) expressed good experiences, 78% (78/100) understood how the intervention works, and 92% (92/100) could use the pillbox, such as opening the device, refilling the pills, and recharging the box (intervention coherence). Of the 100 participants, 84 reported improved adherence (perceived effectiveness), and they preferred to continue receiving reminder SMS text messages (self-efficacy). A few participants reported experiencing difficulties while using DATs. Some participants reported experiencing TB-related stigma; 12% (12/100) were not happy being reminded daily, and 9% (9/100) reported experiencing difficulties keeping the device at home. Additionally, 30% (30/100) reported experiencing challenges with mobile network connectivity issues.

### Comparison With Prior Work

Our findings support similar studies reporting on the acceptability of DATs among patients and health care providers [12,13,15,24]. The potential benefit of DAT, such as its ability to monitor medication adherence or ease of use, was deemed valuable by patients and TB care providers. A study done in South Africa reported that the acceptability of DAT was highly associated with its ease of use among patients [12]. The real-time medication monitoring reports and feedback on adherence helped patients understand their health conditions and led to improved patient and health care provider relationships [12,24,25]. In addition, it led to improved care practice and behavior change among TB care providers. Many health care providers reported feeling more accountable for patient follow-up and motivating patients to adhere to the time of medication intake. Feedback on adherence counseling also generated a sense of care among patients, which had an impact on the psychosocial life of the patients.

However, participants reported several challenges with DATs, such as incorrect sending of SMS text messages due to network failure, large size of the pillbox, and existence of the reminder alarm, which led to fear of disclosure and, consequently, nonuse of the device during travelling. Similar challenges have been reported in other studies [14,26], which, if not well addressed, might contribute significantly to the nonuse of DATs and less uptake of DATs [24]. TB care providers reported increased workload during the use of DATs. Similar findings were reported in a study done in China, where health care providers reported a moderate workload increase during DAT implementation [16]. However, this contradicts a study in India, which reported a decreased health care workload [24]. We found that the increased workload by health care workers was reported as a major concern in settings with shortage of care staff—mainly dispensaries and health centers. Larger facilities such as hospitals reported a slight increase in the workload. In addition, 48% (48/100) of the participants stated that adherence reports from the device were not shown nor utilized in their conversations with health care workers. The health care provider was likely to have little knowledge of the value of adherence reports as a tool for counseling. Regular training should be conducted to reinforce their understanding of the intervention for effective scale-up. Fear of TB stigma and unwanted disclosure should be considered for effective intervention scale-up.

### Limitations and Strengths

This study had certain limitations. One significant limitation was that because of the COVID-19 pandemic, all exit interviews were conducted via phone calls, which posed challenges related to network connectivity and potential interruptions during the questioning process. Mitigation strategies were employed, such as recapping participant responses to ensure accurate information capture. Another limitation was the small sample size in the exit survey compared to the total number of enrolled participants in the trial. The small sample size can be attributed to the public policy implemented in July 2021, wherein unregistered SIM cards were blocked, making it difficult to reach most participants. Nonetheless, we found that the demographic characteristics of the participants who were interviewed (100/245) did not differ from those of the participants who were not interviewed due to the change in the government policy, indicating that the interviewed participants were likely representative of those who were not interviewed. Additionally, 59.2% (145/245) of the participants who were not reached due to the change in the government policy were not affected during the medication period. The change in the government policy regarding SIM card registration occurred when many of our participants had already completed the treatment follow-up and were waiting for the exit survey. In this case, the change in the government policy impacted the exit survey process rather than the intervention itself.

This study has notable strengths that enhance its significance and scope. First, we enrolled participants from all TB-providing facilities in the Kilimanjaro region, thereby offering a comprehensive understanding of acceptability from a broader perspective. Notably, our research pioneers the investigation of the evriMED reminder pillbox’s acceptability among patients with TB in East Africa, providing valuable insights on the acceptance. Additionally, using the TFA facilitated a robust understanding of the acceptability of the evriMED reminder pillbox among patients with TB.

### Conclusion

Our study demonstrates the positive acceptance of a DAT (evriMED) among patients and TB care providers for improving anti-TB drug adherence in Kilimanjaro, Tanzania. Although the potential acceptability of DATs is evident, addressing concerns related to mobile network connectivity and participants’ preferences regarding the number of reminder SMS text messages, and providing adequate training and technical support to health care providers are critical for successful implementation. Future research should explore the impact of evriMED on large-scale implementation in different settings.

## Appendix

Multimedia Appendix 1 EvriMED monitor and its associated intervention.

Multimedia Appendix 2 Exit survey findings.

## Abbreviations

DAT: digital adherence tool
TB: tuberculosis
TFA: theoretical framework of acceptability
