# Supplementary figures and images for: Acceptability of a Digital Adherence Tool Among Patients With Tuberculosis and Tuberculosis Care Providers in Kilimanjaro Region, Tanzania: Mixed Methods Study
Source: Online J Public Health Inform. 2024 Jun 26;16:e51662. doi: 10.2196/51662 (PMC11237791; doi:10.2196/51662)

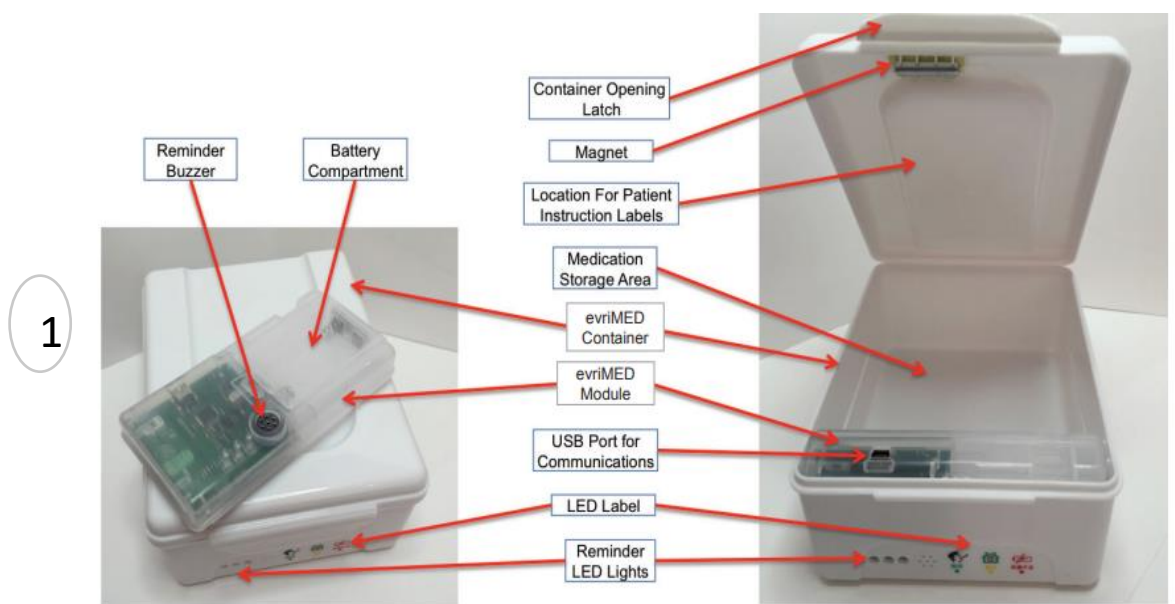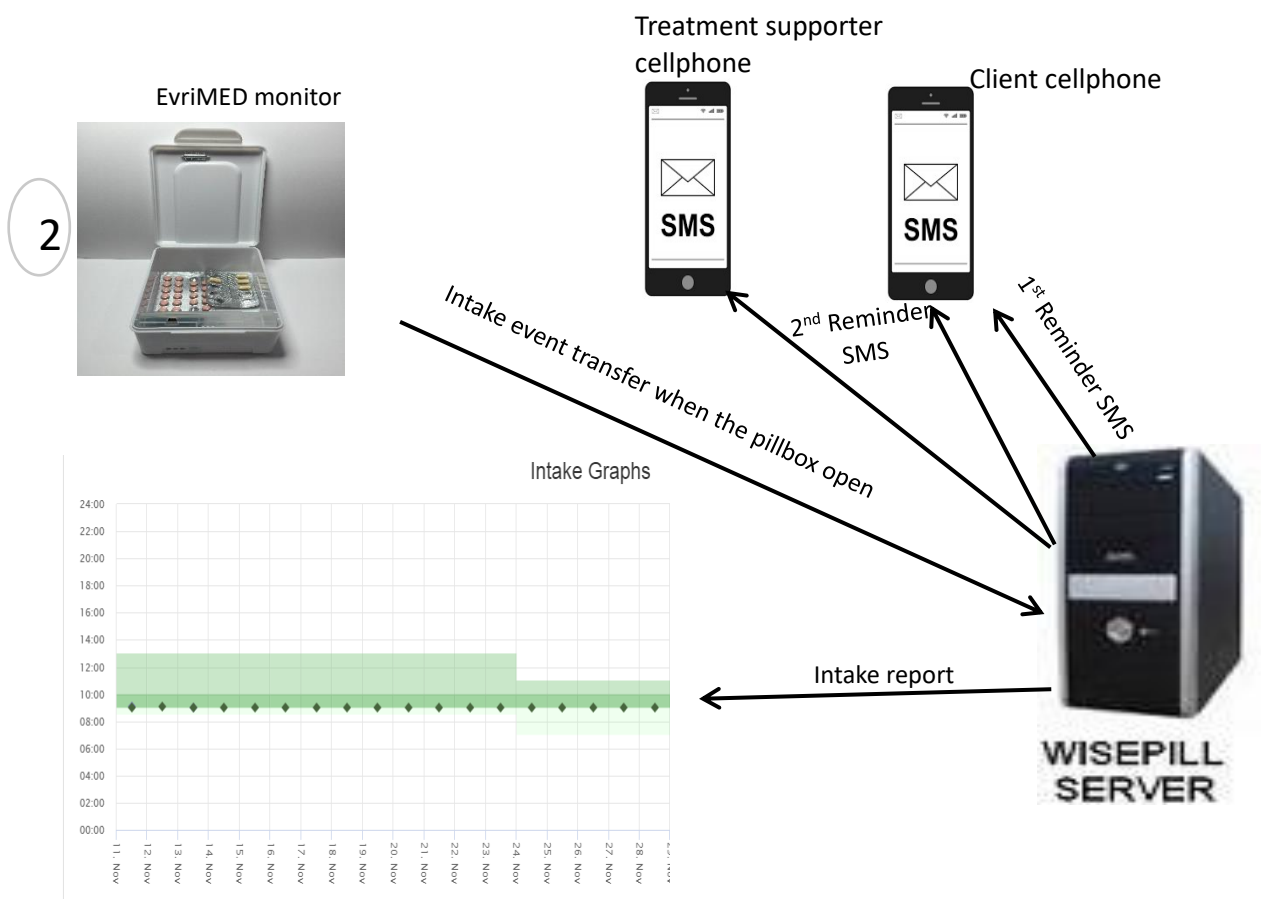

Figure S1: EvriMED Components (1) and the intervention (2)

Supplement: Multimedia Appendix 1 [file ojphi_v16i1e51662_app1.pdf]
