# Supplementary material for: Acceptability of a Digital Adherence Tool Among Patients With Tuberculosis and Tuberculosis Care Providers in Kilimanjaro Region, Tanzania: Mixed Methods Study
Source: Online J Public Health Inform. 2024 Jun 26;16:e51662. doi: 10.2196/51662 (PMC11237791; doi:10.2196/51662)
Supplement: Multimedia Appendix 2 [file ojphi_v16i1e51662_app2.pdf]

Table S1. Quantitative result on Patients' acceptability of DATs

Acceptability Constructs (derived from the questionnaire)	Value (%), N=100
**Affective attitude**	
1. Participants general experience of using intervention	
Good/very good	98(98)
Not good	2(2)
2. Patients satisfied with the intervention	
Yes	83(83)
No	5(5)
3. Participants attitude with the Content of reminder SMS	
Good/very good	85(85)
Not good	15(15)
**Ethicality**	
1. Participants experienced any form of stigma while using intervention	
Yes	10(10)
No	77(77)
2. Participants worried being remotely monitored	
Yes	3(3)
No	8(85)
**Perceived Burden of the intervention**	
1. Difficult using intervention	
Yes	7(7)
No	85(85)
2. Participant experience TB related stigma	10(10)
Yes	
No	77(77)
3. Participants comfortable receiving reminder SMS	
Yes	84(84)
No	12(12)
4. Difficult storage	
Yes	9(9)

No	82(82)
5. Challenges of internet connectivity	
Yes	30(30)
No	61(61)
6. Difficult charging	
Yes	3(3)
No	83(83)
**Intervention coherence**	
Participants understand the intervention and how it works	
Yes	78(78)
No	18(18)
**Opportunity cost**	
Participants incurred extra cost while using intervention	
Yes	6(6)
No	90(90)
**Perceived effectiveness**	
The intervention improved adherence	
Yes	84(84)
No	3(3)
**Self-efficacy**	
Participants are comfortable to continue receiving reminder SMS	
Yes	84(84)
No	12(12)
